# Supplementary material for: Anionic Nanoplastic Contaminants Promote Parkinson’s Disease-Associated α-Synuclein Aggregation
Source: Res Sq. 2023 Oct 13:rs.3.rs-3439102. Preprint. [Version 1] doi: 10.21203/rs.3.rs-3439102/v1 (PMC10602106; doi:10.21203/rs.3.rs-3439102/v1)
Supplement: Supplement 1 [file NIHPPRS3439102V1-supplement-1.pdf]

Supplementary Materials for

Anionic Nanoplastic Contaminants Promote Parkinson's Disease-Associated α-Synuclein Aggregation

Zhiyong Liu*[1], Arpine Sokratian*[1], Addison M. Duda[2], Enquan Xu[1], Christina Stanhope[1], Amber Fu[1], Samuel Strader[1], Huizhong Li[1], Yuan Yuan[1], Benjamin G. Bobay[2], Joana Sipe[3], Ketty Bai[1], Iben Lundgaard[4,5], Na Liu[4,5], Belinda Hernandez[6], Catherine Bowes Rickman[6], Sara E Miller[7], Andrew West[1]**

**Corresponding author: Email: andrew.west@duke.edu

Figs. S1 to S14
Captions for Figs. S1 to S14
Table S1
Supplemental Methods
Captions for Auxiliary Supplemental Materials

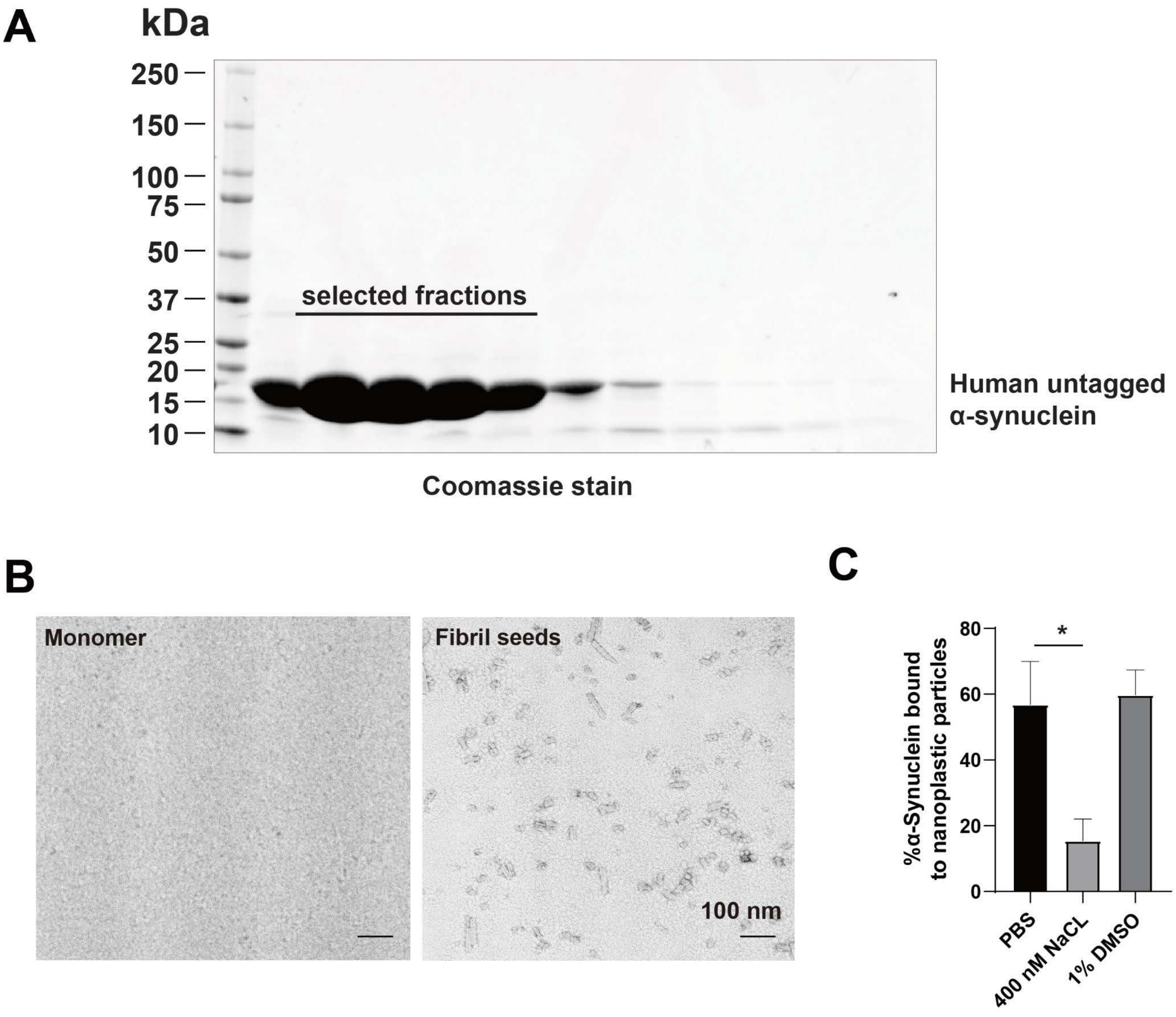

Fig. S1. Validation of monomeric and α-synuclein fibril seeds.

A) Representative SDS-PAGE and Coomassie stain of selected eluted fractions from boiled bacterial lysates with untagged human α-synuclein protein processed through HiPrep Q HP 16/10 columns.

B) Representative negative stain electron microscopy of purified α-synuclein monomer and fibril seeds (generated by sonication of full length fibrils to the terminal stage). Monomer protein was screened via TEM to detect possible oligomers or larger aggregates. Processed sonicated fibril seed length was consistent with light-scattering diameter estimations for the stumpy fibril particles. Very small stumpy fibril particles are exceptionally stable under physiological conditions according to past analyses (*24*). Scale bar, 100 nm.

C) Group analysis of the propensity of α-synuclein fibrils to bind nanoplastic particles after 30 min RTe incubation in PBS, or PBS supplemented with 400 nM NaCl (i.e., high-salt), or 1% DMSO in water, measured by light-scatter. Each group mean (column) represents an average of 10 acquisitions with error bars indicating SEM.

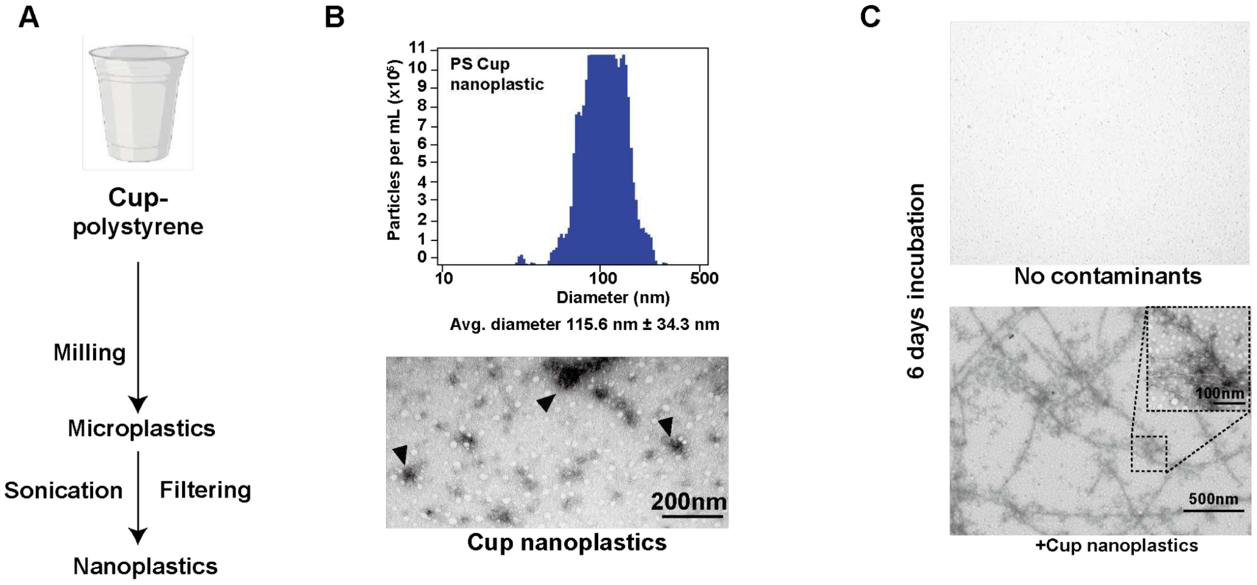

Fig. S2. Cup-abraded environmental polystyrene nanoplastic in α-synuclein aggregation.

A) Workflow of nanoplastic production from plastic cups. Microplastics from polystyrene plastic cups generated by grinding were sonicated to break larger aggregates and then filtered (0.2-micron) to enrich nanoplastic particles.

B) Representative light scattering plot and TEM of abraded nanoplastics contaminants from a polystyrene cup. Arrowheads indicate particles with sizes consistent with light scattering analysis (upper panel, blue). Scale bar, 200 nm.

C) Representative TEM images of 70 μM human α-synuclein (monomeric) shaken at 37 °C in absence (top) or presence of 1 nM (estimated, Zeta nanoparticle tracking analysis) polystyrene cup nanoplastic contaminants for 6 days. Bounding boxes show magnified representative fibrils and nanoplastic-tethered particle interactions. Bars = 500 nm and 100 nm for magnified inset.

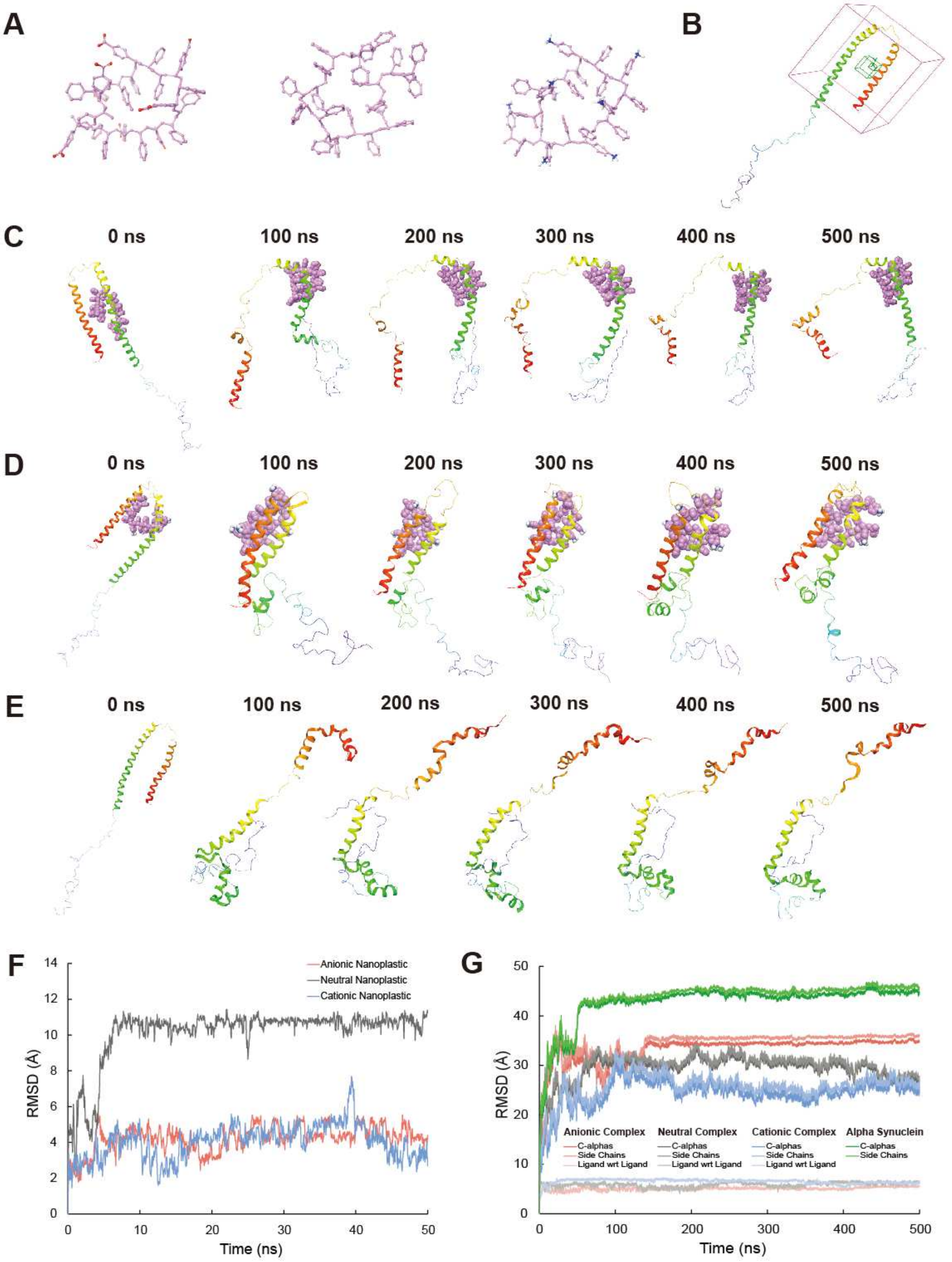

Fig. S3. Structures and molecular dynamics time frames, ligand RMSDs, and complex RMSDs that highlight the differential effects of anionic nanoplastics on α-synuclein folding.

A) Representative frames of converged geometry-optimized anionic, neutral, and cationic nanoplastics, left to right (ball-and-stick, only polar hydrogens shown; white = hydrogen, pink = carbon, blue = nitrogen, red = oxygen).

B) Representation of docking box (purple, 46 Å) used for the Induced Fit Docking method to introduce nanoplastics to α-synuclein monomeric protein.

C) Snapshots of neutral nanoplastic and α-synuclein (ribbon colored by residue index) complex, D) cationic nanoplastic and α-synuclein complex, or E) α-synuclein alone across MD simulations, at 100 ns intervals. Nanoplastics depicted as space fill, showing polar hydrogens only; white = hydrogen, pink = carbon, blue = nitrogen. α-Synuclein is depicted in ribbons and colored by residue index. Of note, neutral and cationic nanoplastics have different associations, fewer contacts, and weaker interactions with α-synuclein as compared to anionic nanoplastic.

F) RMSD plots of MD-simulated nanoplastics confirming simulations converge within 10 ns. RMSDs were calculated for heavy atoms of each ligand using frame 1 as a reference.

G) RMSD plots of nanoplastic/α-synuclein complexes and α-synuclein alone confirming simulations converge within 200 ns. RMSDs were calculated for indicated atoms of the complexes and α-synuclein alone using frame 1 as a reference. C-alphas is the RMSD of only the α-carbons of α-synuclein's backbone, Side Chains is the RMSD of only the heavy atoms of the residue side chains. Ligand with respect to (wrt) Ligand is the RMSD calculated for the heavy atoms of the nanoplastic in the α-synuclein complex. Simulations converge within 200 ns.

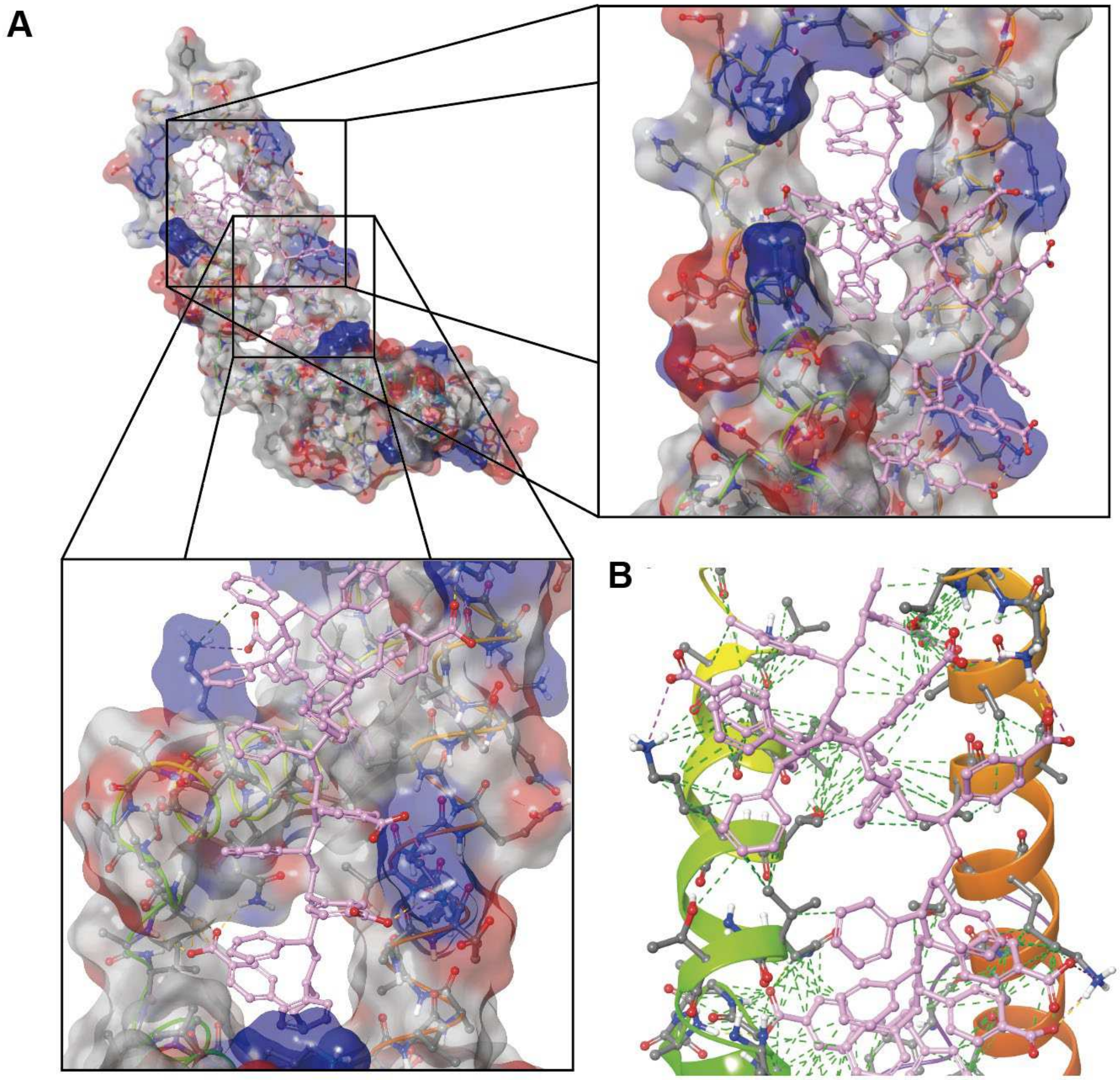

Fig. S4. Anionic nanoplastic complex molecular surface and Van der Waals (VDW) contacts highlight water displacement and tight association.

A) Representative frame of anionic nanoplastic and α-synuclein complex, with individual blow ups highlighting plastic-protein surface interactions. Carboxylates associate with areas of positively charged densities to form strong polar bonds while the hydrophobic backbone of the nanoplastic occupies regions rich with nonpolar residues. The surface of α-synuclein bound to anionic nanoplastic becomes free of solvent water. Anionic nanoplastic is depicted as ball-and-stick, showing polar hydrogens only; pink = carbon, red = oxygen. α-synuclein backbone depicted as cartoon ribbon colored by residue index; residues are ball-and-stick showing polar hydrogens only; white = hydrogen, grey = carbon, blue = nitrogen, red = oxygen; 50 % transparent molecular surface colored by residue charge, blue = positive, grey = neutral, red = negative

B) A representative web of Van der Waals (VDW) contacts shows tight association of the anionic plastic in the protein complex. Shown is a matched frame (as in Figure 2D), but now indicating VDW interactions. Anionic nanoplastic forms a web of good VDW contacts with α-synuclein. Green dashed lines represent good VDW contacts, whereas orange dashed lines represent rough VDW contacts, and red designates bad VDW contacts.

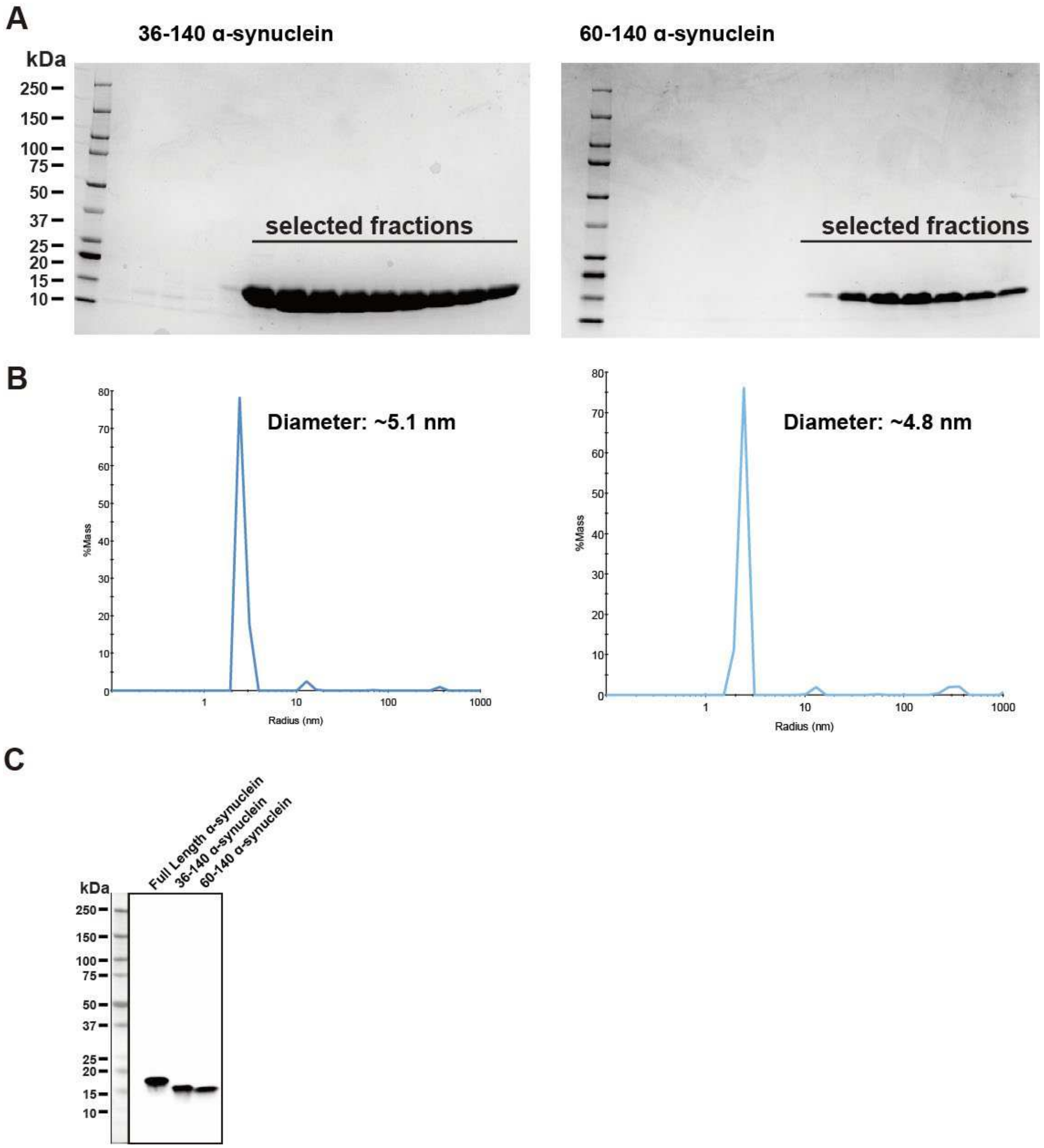

Fig. S5. Validation of N-term truncated α-synuclein recombinant proteins.

A) N-truncated α-synuclein variants 36-140 and 60-140 were prepared similarly to full length protein. Representative SDS-PAGE and Coomassie stain analysis of eluted fractions demonstrate pure protein preparations selected for further purification and concentration. Variant 36-140 shows lower molecular weight as expected, with a negative charge of -11.2 at pH 7.5, while 60-140 has a negative charge of -12.2 at pH 7.5.

B) Representative dynamic light scattering (DLS) histograms of purified 36-140 (left) and 60-140 (right), plotted relative to the mass distribution-predicted molecular weight, demonstrating monomeric (i.e., non-aggregated) protein.

C) Representative immunoblot to detect (~1 ng loaded each lane) recombinant full length (1-140) and truncated variants (36-140 and 60-140) with the monoclonal anti-α-synuclein antibody MJF1R that binds nearer the C-terminus.

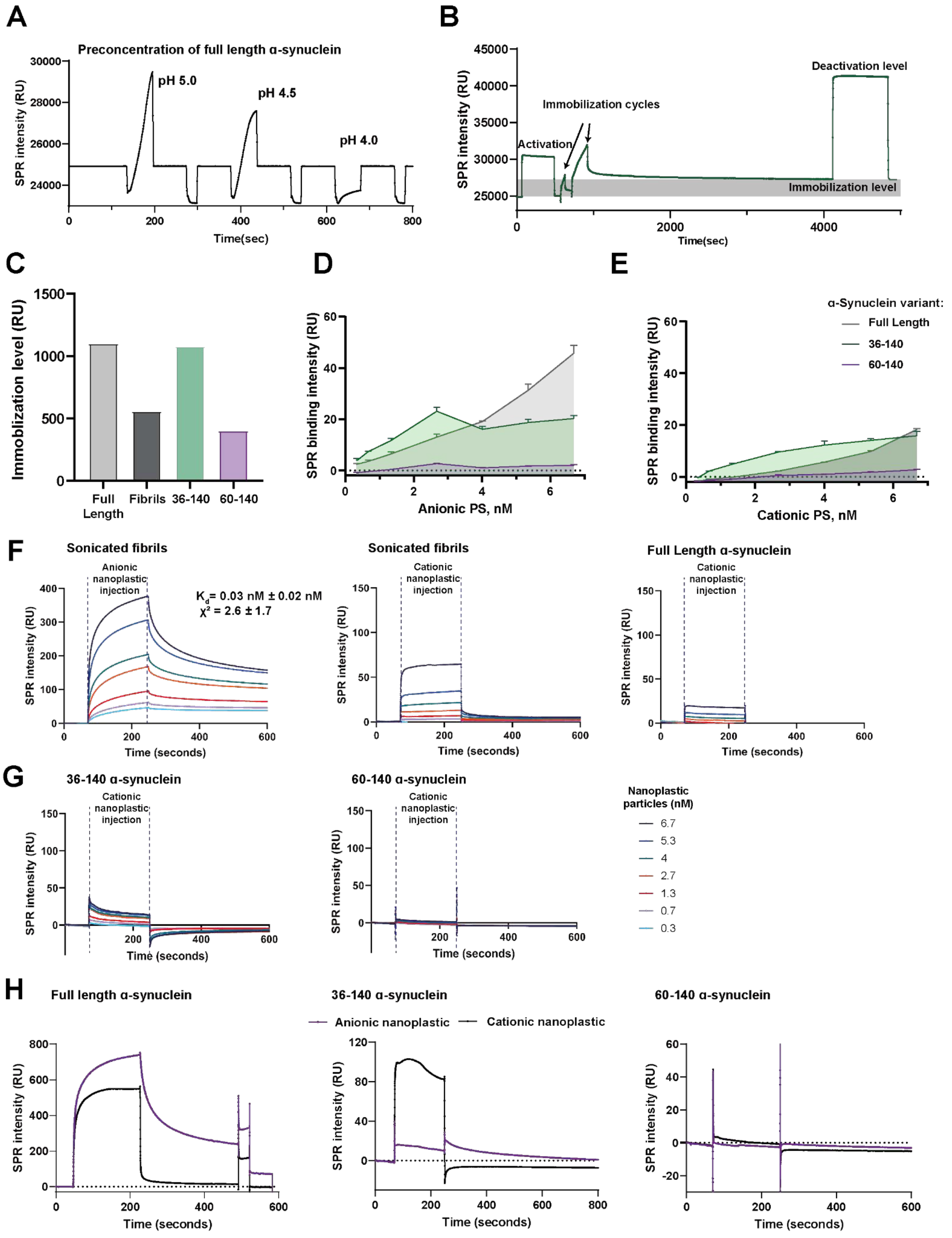

Fig. S6. Surface plasmon resonance analysis showing activation, immobilization, deactivation, and the necessity of negatively-charged nanoplastic for interaction with the amphipathic domain in α-synuclein.

A) A representative graph of a pre-concentration SPR sensorgram indicates the effects of immobilization pH (10 mM sodium acetate) leading to a change of the electrostatic attraction of α-synuclein at the surface of the CM5 chip resulting in a spike of SPR intensity. Dilution of protein in the sodium acetate buffer at different pH allows for the identification of efficient immobilization conditions. Exemplary injections of full length α-synuclein at different immobilization pH indicate the highest spike of SPR intensity at pH 5 with gradual decreases from pH 4.5 to 4.

B) Representative SPR sensorgram of α-synuclein immobilization to an SPR chip activated by 1-ethyl-3-(3-dimethylaminopropyl) carbodiimide (EDC)/N-hydroxysuccinimide (NHS), enabling amine-targeted immobilization. Deactivation with ethanolamine quenches any unreacted carboxyl groups and rids the surface of nonspecifically bound α-synuclein. Increases in SPR intensity after iterative cycles of immobilization, highlighted in gray, indicates the effects of conjugation of α-synuclein to the chip with 200 - 1000 RU, an index generally compatible with sensitive kinetic measurements.

C) Columns depict final immobilization as shown in B) for full length α-synuclein, α-synuclein fibrils, and the two N-terminal truncated variant proteins.

D) Group analysis histograms of changes in SPR binding intensity upon injection of anionic and E) cationic nanoplastic particles for interaction with full length α-synuclein, and the truncated 36-140 and 60-140 variant proteins. The rate of binding in SPR measurements was calculated with Biacore T200 Evaluation Software as SPR RU at the end of the analyte injection, with subtracted blank reference values.

F) Titration-based SPR curves of immobilized α-synuclein fibrils with injection of nanoplastic particles demonstrates anionic (left panel), but not cationic nanoplastic, can interact tightly with α-synuclein. Extracted average K_d values from three independent reactions with indicated SEM and X_2, calculated with heterogeneous analyte model fit on Biacore T200 software. Binding analyses of both fibrils (middle) and full length monomeric α-synuclein protein (right panel) to cationic nanoplastic particles were marked as negative in the fitting process. While addition of cationic nanoplastic to fibrils immobilized on the chips results in spikes of intensity during injection steps, dissociation cycling leads to immediate return to the initial RU levels, indicative of weak and non-specific interaction.

G) Binding analysis of cationic nanoplastic to 36-140 (left) and 60-140 (right) α-synuclein variants measured by SPR kinetic analysis.

H) Representative SPR sensorgrams of interactions between immobilized full length α-synuclein (left), truncated 36-140 protein (middle), and truncated 60-140 protein (right) and anionic or cationic nanoplastic at 15 nM. Anionic nanoplastic particle injection shows specific association and dissociation profiles exclusively in the presence of conjugated full length α-synuclein, while positively charged particles demonstrate strong associations followed by rapid dissociations that highlight weak and non-specific interactions. Truncated 36-140 α-synuclein shows weak association with cationic particles and no response with anionic nanoplastic. Likewise truncated 60-140 protein demonstrates no significant binding to anionic or cationic nanoplastic particles.

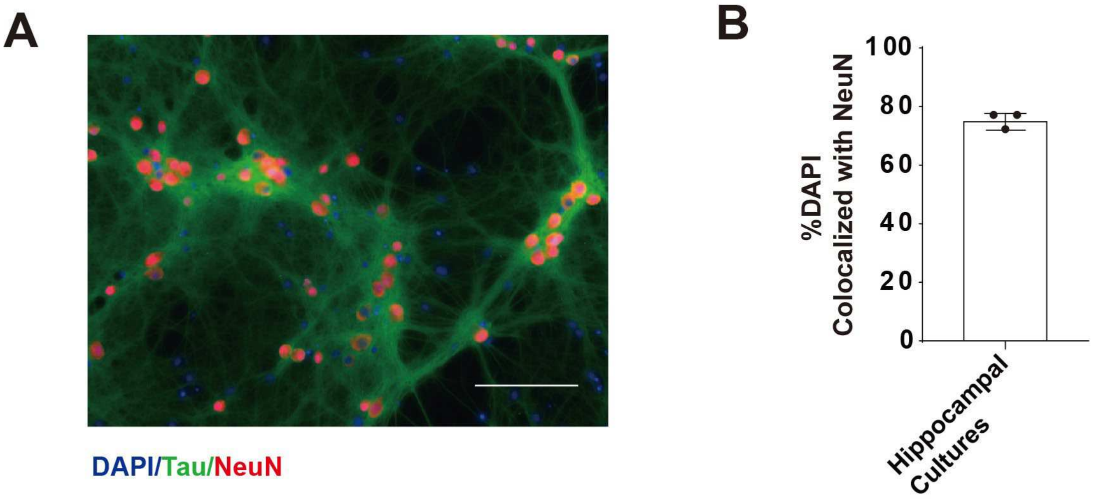

Fig. S7. Characterization of primary neurons via tau and NeuN immunostaining shows high neuronal purity and high neuronal tau-positive process complexity.

A) A representative image of primary mouse hippocampal neurons used in this study, derived from post-natal (P0) CD-1 mice and cultured for at least 7 days *in vitro* (DIV 7) before treatment with nanoplastics (or fibrils) as indicated. Representative images show highly arborized and interconnected neurons. Red indicates NeuN staining that primarily localizes to the neuronal cell body, and blue labels DAPI staining primarily localized to the nucleus, and green shows tau staining distributed smoothly along neuritic processes.

B) The ratio of Hoechst-positive to NeuN-positive cells (e.g., neurons) in cultures (from N=3 biologically independent experiments, SEM) shows a minority of non-neuronal cells in culture. The NeuN-negative cells are presumed to be primarily astrocytes that are morphologically distinct from neurons in the culture.

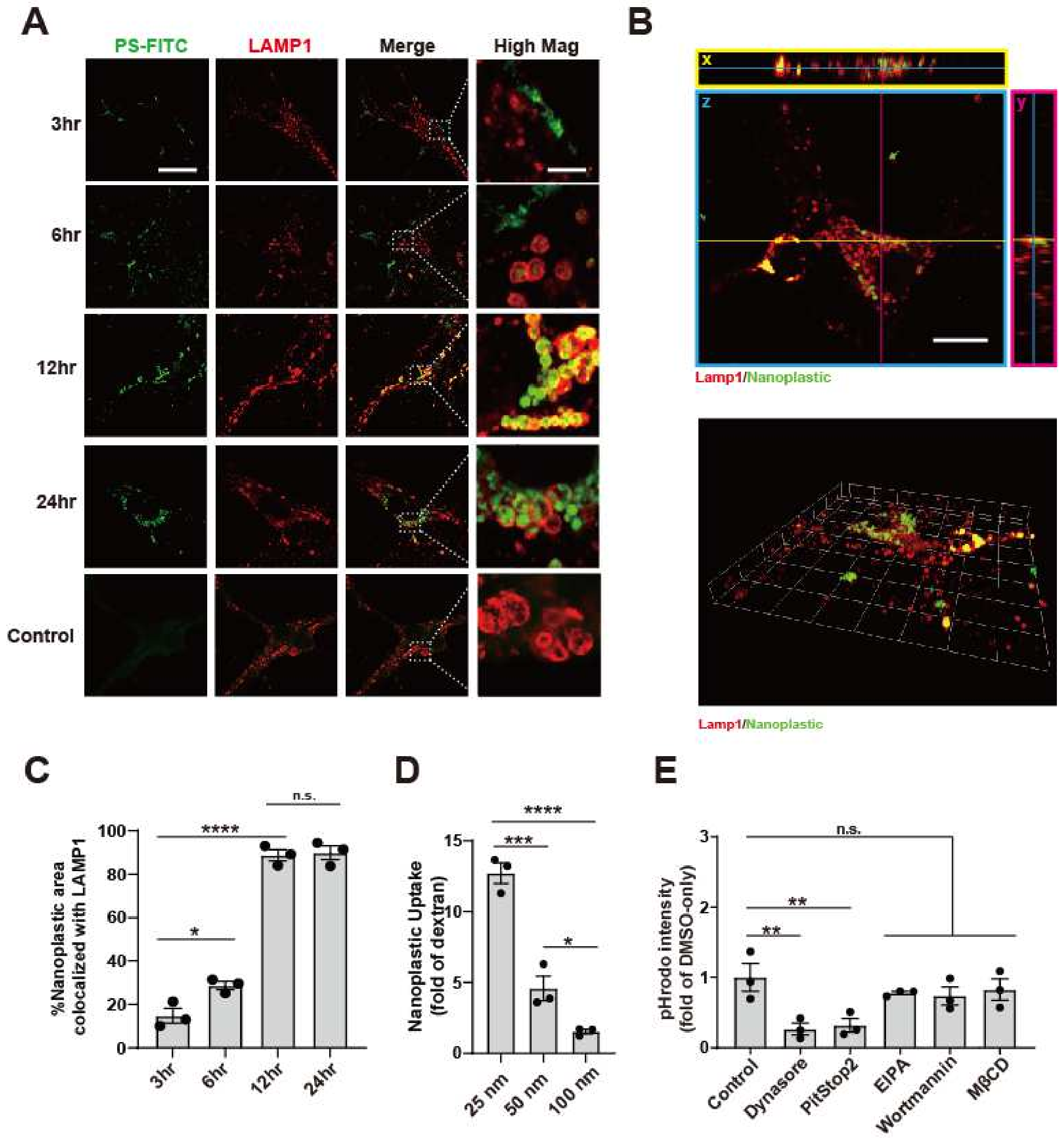

Fig. S8. Primary mouse hippocampal neurons endocytose nanoplastics through a dynamin-dependent process.

A) Representative confocal Airyscan images from mouse primary hippocampal neurons (DIV 7) treated with FITC-labeled (green) nanoplastic (1 nM) for 3, 6, 12 and 24, hrs. Scale bar, 10 µm and 1 µm for "High Mag".

B) A representative Airyscan confocal image from mouse primary hippocampal neurons (DIV 7) simultaneously exposed to FITC-labeled nanoplastics. Neurons were immuno-stained at 24 hours after the addition of the particles. The orthogonal view (left panel) from sequential Z-stacks is shown. Pink line is the intersection of y,z; Yellow line is x,z;

Cyan line is x,y. Cyan box is the x,y plane; Pink box is the y,z plane; Yellow box is the x,z plane. Scale bar represents 10 µm. The reconstructed 3D view is shown in the right panel.

C) C-E) Data corresponding to main Fig. 4 panels B,D, and F are shown where each dot is the mean value from the analysis of all images from three biologically independent experiments. Each column shows the group mean (SEM). $*p < 0.05$, $**p < 0.01$, $****p < 0.001$, and n.s. is not significant, one-way ANOVA and Tukey's post hoc test.

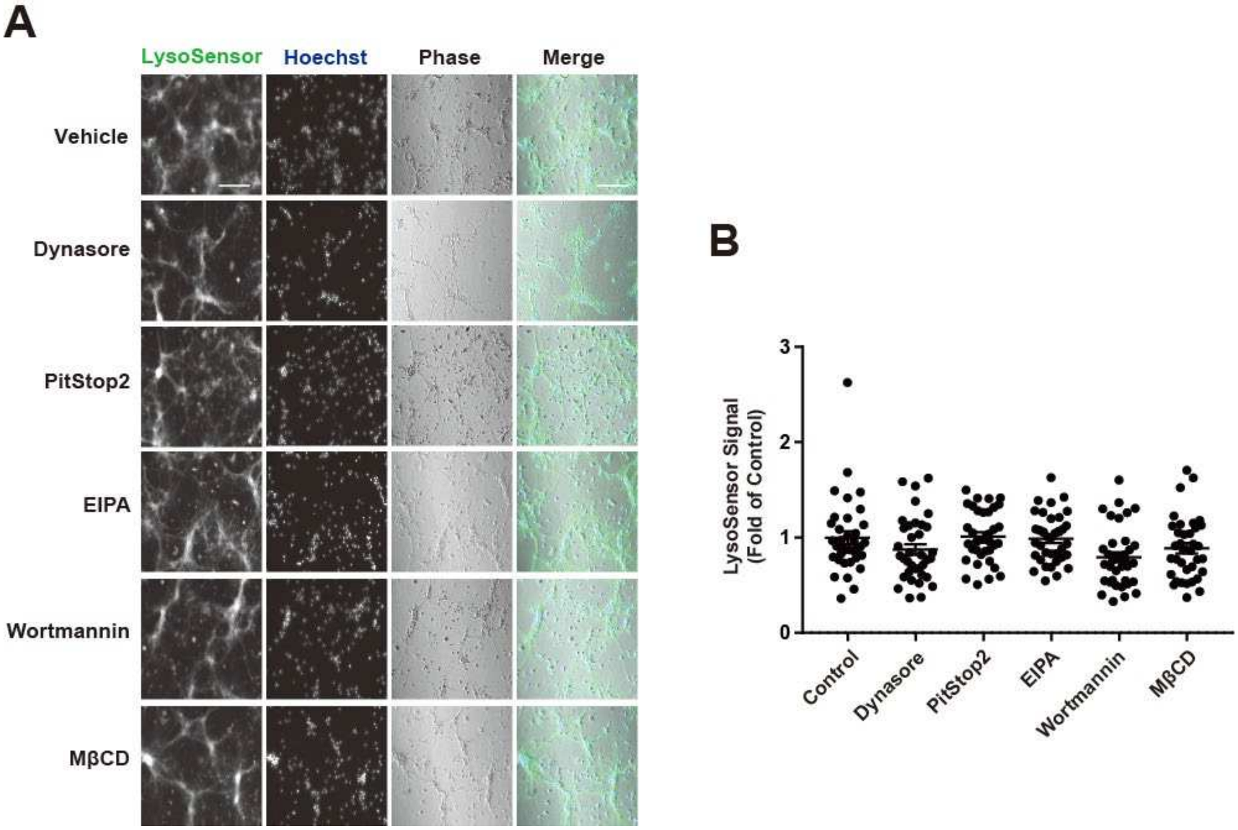

Fig. S9. Endocytosis inhibitors in neurons do not result in acute lysosomal acidification differences within the 2-hour timeframe used here for analysis.

A) Widefield microscopy of primary hippocampal neurons at DIV 7, stained with 1 μM LysoSensor (green) or Hoescht dye, shows similar neuronal morphologies and LysoSensor intensities across different conditions. Vehicle, or the indicated inhibitor, was added two hours prior to incubation with 1 μM LysoSensor Green for 30 minutes. Cells were washed two times with Hank's Balanced Salt Solution (HBSS) before staining with Hoescht dye. Scale bar, 200 μm.

B) Quantification of relative LysoSensor green (i.e., lysosomal acidity) calculated as a fold-change from DMSO-only (control) treated cells. Twenty-seven images from three independent experiments were quantified for each condition, with each dot representing results from one image. Bars show group means (SEM), and one-way ANOVA analysis was not significant.

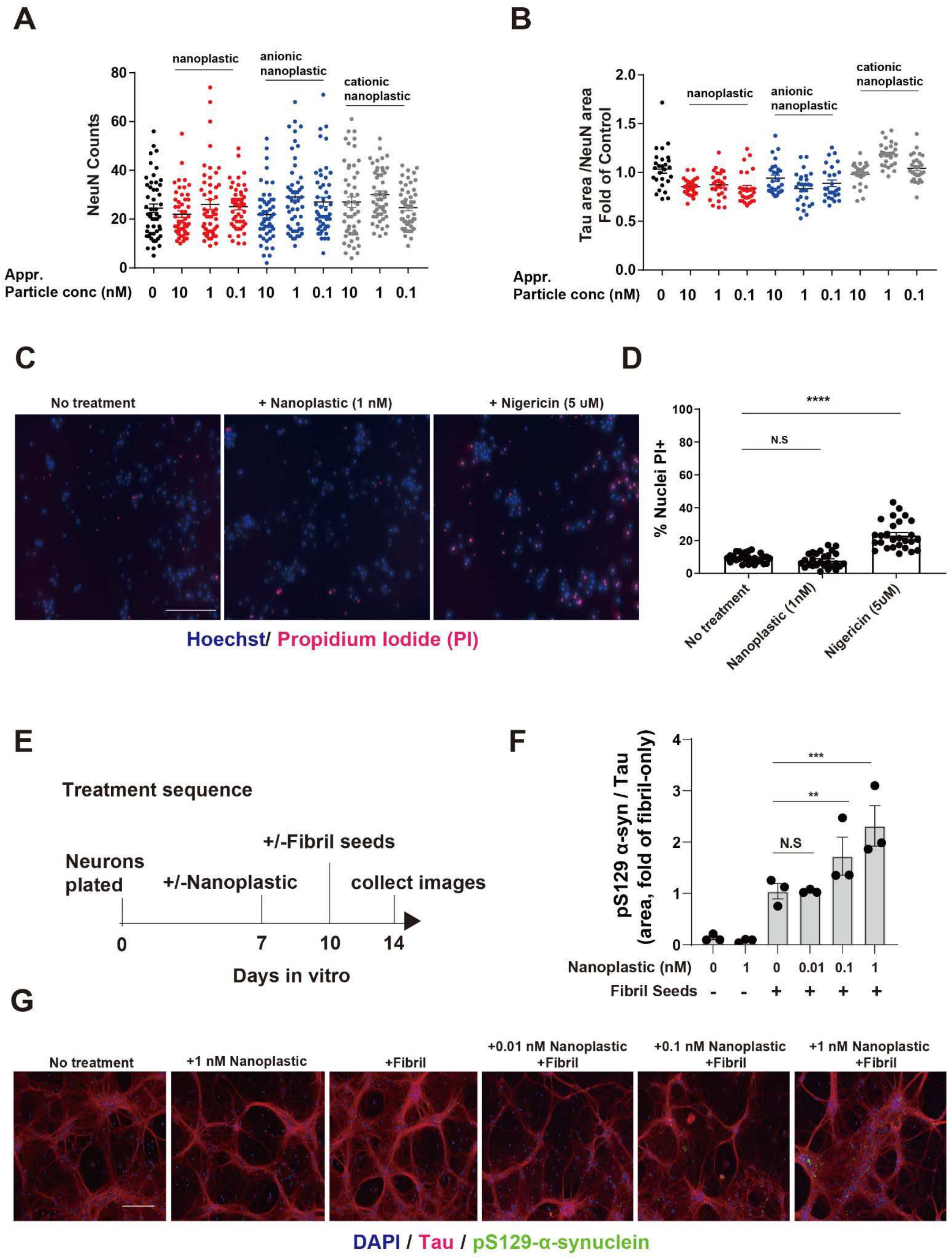

Fig. S10. Low neuronal toxicity associated with nanoplastic exposures to primary neurons, and effects of different nanoplastic concentrations on fibril-seeded α-synuclein aggregation.

A) Primary hippocampal neurons (DIV 7) were treated with the indicated concentration of nanoplastic particles, and column plots show image field counts for the number of NeuN-positive cells. The average number of NeuN-positive cells per field is quantified from fifty images collected from three independent experiments, where each dot represents the analysis of one image.
B) Relative ratios of tau-positive areas to NeuN are calculated as a measure of neuronal process complexity. Each dot shows the analysis of one image of twenty-seven images from three independent experiments. Black bars are group means (SEM) in graphs in A) and B). There were no apparent losses of NeuN positivity of tau complexity with the nanoplastic concentrations indicated.
C) Representative fluorescence from neurons exposed to 1nM of anionic nanoplastic, or neurons treated with 5 μM nigericin for 2 hrs (an NLRP3 activator and mild neurotoxin), prior to staining with propidium iodide and Hoescht.
D) The percentage of Hoechst nuclei that are co-positive for propidium iodide are evaluated as a measure of cell death in neurons after treatment with nanoplastic or nigericin. Dots are the mean values from each image of 27 images collected from 3 independent experiments. Black bars show group means (SEM), with significance assessed by one-way ANOVA with Tukey's post hoc test. ****$p<0.0001$, and n.s. is not significant.
E) Graphical timeline for the sequential addition of nanoplastic (or vehicle control, at DIV 7) and then α-synuclein fibrils (at DIV 10), with neurons immunostained and imaged at DIV 14.
F) Quantification of pS129-α-synuclein area occupying tau-positive area as a measure of the abundance of α-synuclein pathology. Each dot represents the combined (mean) analysis of images from an independent experiment. Black bars show group means (SEM), with significance assessed by one-way ANOVA with Tukey's post hoc test. **$p<0.01$, ***$p<0.001$ and n.s. is not significant.
G) Representative images of neurons treated with nanoplastics and then α-synuclein fibrils. Immunofluorescence images for tau and pS129-α-synuclein at DIV 14 are shown. Scale bars, 200 μm.

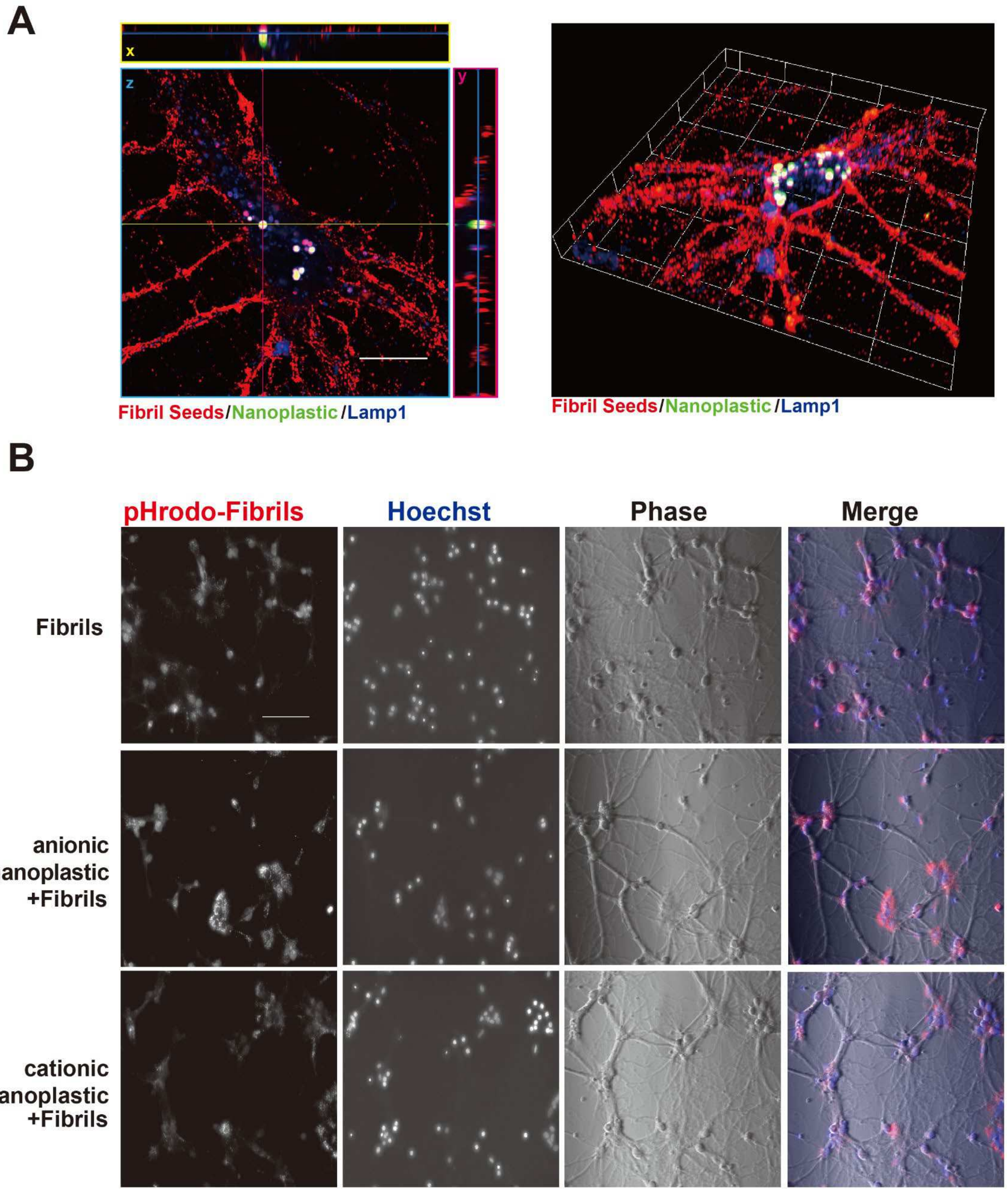

Fig. S11. Nanoplastics do not alter the initial internalization of α-synuclein fibrils into acidified vesicles in neurons.

A) A representative Airyscan confocal image from mouse primary hippocampal neurons (DIV 7) simultaneously exposed to both FITC-labeled nanoplastics and Alexa-647-labeled human α-synuclein fibrils. Neurons were immuno-stained at 24 hours after the

addition of the particles. The orthogonal view (left panel) from sequential Z-stacks is shown. Pink line is the intersection of y,z; Yellow line is x,z; Cyan line is x,y. Cyan box is the x,y plane; Pink box is the y,z plane; Yellow box is the x,z plane. Scale bar represents 10 μm. The reconstructed 3D view is shown in the right panel.

B) Representative images from widefield microscopy of neurons with 1 nM of either anionic or cationic polystyrene beads added for 2 hours prior to the addition of 66.7 pM pHrodo-labeled α-synuclein fibrils. Cells were washed two times with Hank's Balanced Salt Solution (HBSS) to help eliminate non-internalized particles prior to the cultures stained with Hoechst. Further, the dye pHrodo-Red should not fluoresce at all until internalized in cells into an acidified vesicle. An analysis of >50 images analyzed for each condition suggests the nanoplastic addition does not change the uptake of α-synuclein fibrils into neurons. Scale bar, 200 μm

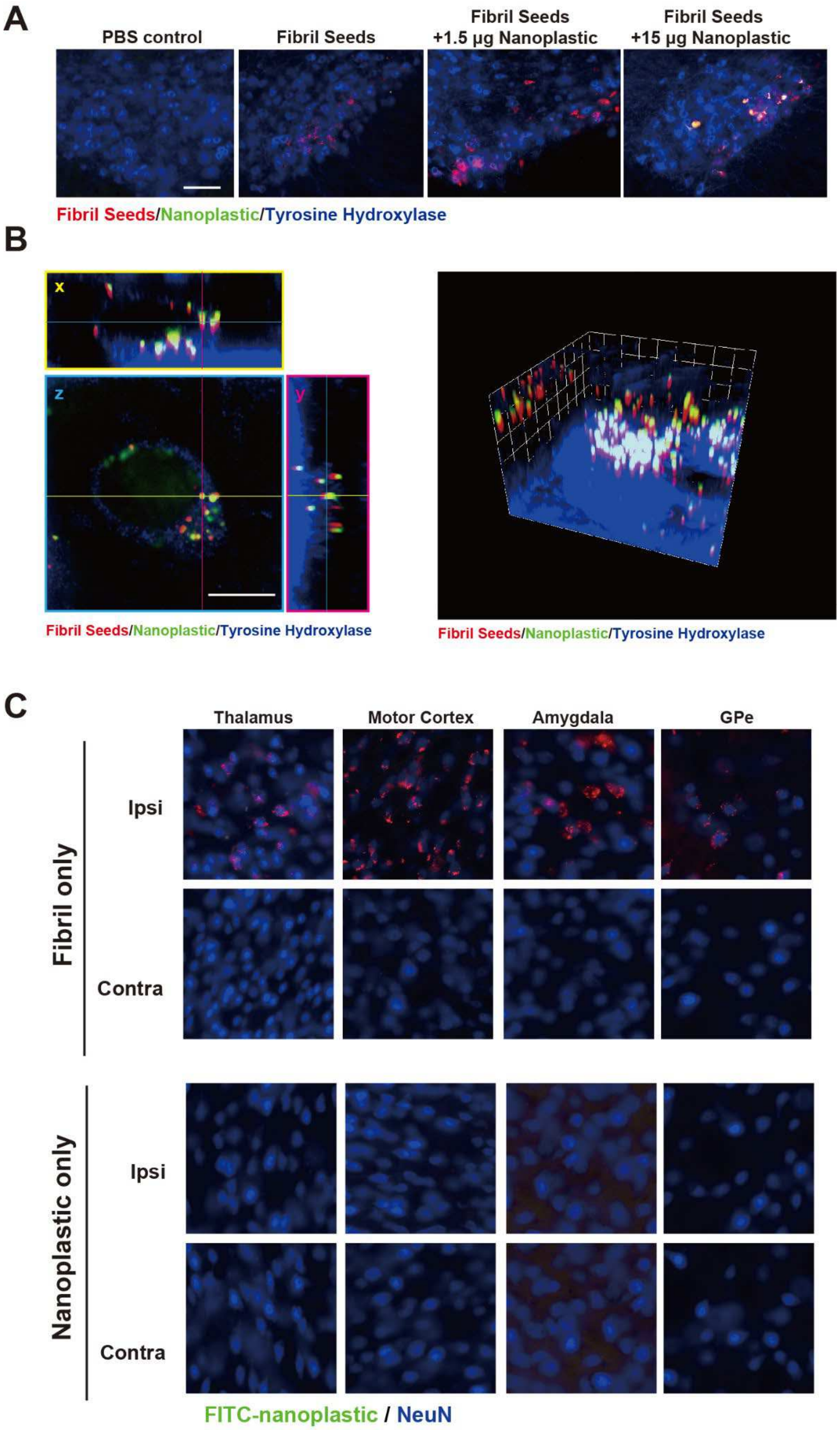

Fig. S12. Fibril or nanoplastic spread through the mouse brain 3-days post intrastriatal injection.

A) P90 male CD1 (non-transgenic) mice were injected unilaterally in the dorsal striatum with PBS buffer control, or 4.5 µg Alexa-647 labeled human α-synuclein fibrils, in combination with the indicated amount of FITC-conjugated nanoplastic particles. The

mouse brains were collected for analysis 3-days post-injection. Representative images of dopaminergic cells in the SNpc are shown, with fibril-only (red) or nanoplastic-only (green) injections with tyrosine hydroxylase staining (blue). Scale bar, 50 µm.

B) Orthogonal view (left panel) from sequential Z-stacks is shown from a dopaminergic neuron in the SNpc. Pink line is the intersection of y,z; Yellow line is x,z; Cyan line is x,y. Cyan box is the x,y plane; Pink box is the y,z plane; Yellow box is the x,z plane. Scale bar represents 10 µm. The reconstructed 3D view is shown in the right panel.

C) Additional representative images in the indicated brain region in mice injected with either nanoplastic particles alone or fibril particles alone. Efficient retrograde (or anterograde) spread of FITC-conjugated anionic nanoplastics (green) was not observed in contrast to labeled fibrils. Images are representative of the brain regions from six injected mice that were analyzed. Scale bar, 50 µm.

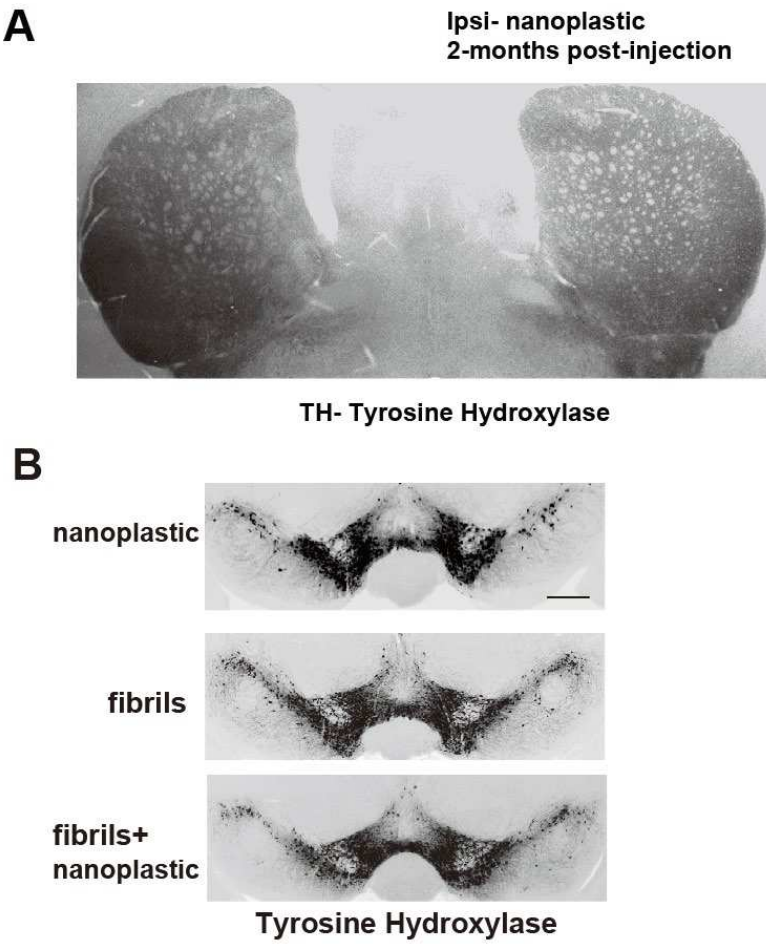

Fig. S13. Striatal nanoplastic or human α-synuclein fibril injections do not result in loss of dopaminergic neurons 2-months post injection.

A) Representative image of fluorescence microscopy of tyrosine-hydroxylase (TH)-stained (grayscale) dorsal striatum. Shown is a representative coronal section near the (coronal) plane of particle injection of an adult mouse 2-months post-nanoplastic injection. No obvious changes in TH+ fiber density were observed, suggesting intact nigrostriatal projections months following nanoplastic injection.
B) Representative images of fluorescence microscopy of tyrosine-hydroxylase (TH)-stained (grayscale) substantia nigra pars compacta (SNpc). striatum. Shown is a representative coronal section near the plane of injection of an adult mouse 2-months post-nanoplastic injection. No obvious changes in the SNpc were observed in TH+ cell distribution and density, suggesting preservation of these neurons (i.e., minimal or no neuronal loss) months following nanoplastic injection, or nanoplastic with fibril injections. Scale bar, 500 μm.

A

ID	Sex	Age	PMI (hrs)	Pathological Diagnosis
1	F	82	1	McKeith DLB Neocortical
2	M	84	17	McKeith DLB Neocortical
3	F	75	16	McKeith DLB Neocortical
4	F	81	15	No pathological changes
5	M	82	10	No pathological changes
6	F	85	13	No pathological changes

B

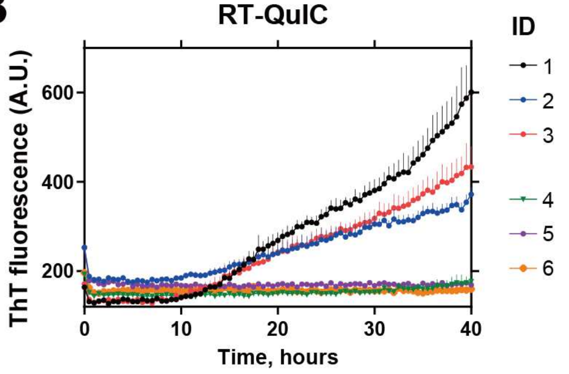

C

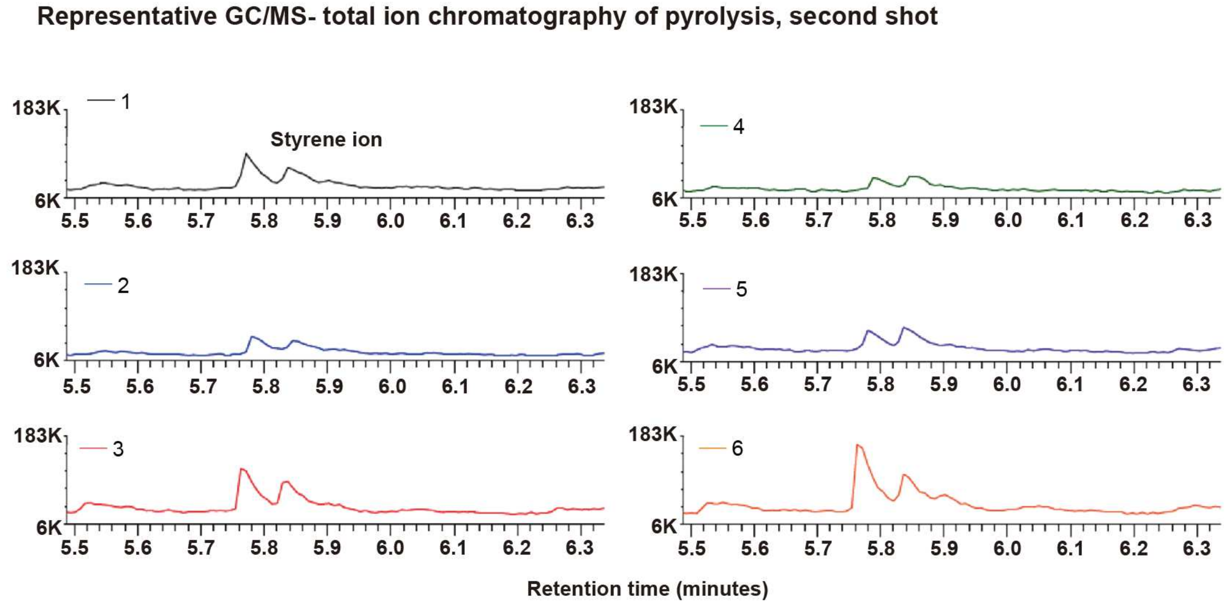

Fig. S14. Detection of styrene ions from frontal cortex brain tissue in Lewy body disease and healthy control brain tissue samples via a double-shot pyrolysis-gas chromatography / mass spectrometry analysis.

A) Six frozen frontal-cortex brain samples were procured from the Bryan Brain Bank at Duke University that correspond to a pathological diagnosis of neocortical dementia with Lewy bodies or neurologically normal. Sex, age at death, post-mortem interval for freezing the brain samples, and pathological diagnosis are indicated.

B) Results from an α-synuclein seeding assay (real time quaking-induced conversion, RT-QuIC) from the frozen frontal-cortex brain tissue piece. These six brain samples were previously analyzed as part of a larger RT-QuIC-based characterization of brains from the Bryan Brain bank as previously reported (*24*).

C) Representative ion chromatogram traces (from duplicate runs) from the analysis of 200 micrograms of desorbed (250°C, 5 min) lyophilized brain tissue fine-powder (blood vessels removed) from the indicated sample. The desorbed product elucidates semi-volatile analytes, whereas the pyrolysis step generates pyrolyzates otherwise stable to

600°C (e.g., polystyrene). A zoomed retention time for the pyrolyzates is shown highlighting peaks associated with styrene ions. All six brain samples were positive for styrene ions. Methods used for this analysis were based on a previous method of the analysis of whole blood samples from healthy controls for the presence of polystyrene nano- and microplastic polystyrene detection (*8*). More details for this method can be found in Supplementary Methods.

Table S1. Source and types of nanoplastics used

Catalog #*	Average diameter- nm (SEM)**	Charge and modification***	Fig.#
PST25C	23.2 (3.9)	Anionic (-COOH)	Fig.1C-H, Fig.3B-D, Fig.5E-H, Fig.6C-K, Fig.7A-E, Fig.9, Fig.S1A, Fig.S6D,F,H, Fig. S7, Fig.S10A-G, Fig.S11C, Fig.S13
PST50C	39.5 (0.7)	Anionic (-COOH)	Fig.1A-B
PST25A	23.5 (1.9)	Cationic (-NH2)	Fig.3B, Fig.S6E-H, Fig.S10D-E, Fig.S11C
FGP25C	36.6 (0.6)	Anionic (-COOH), FITC	Fig. 4A-B, Fig.5A-C, Fig.6A-B, Fig.7D, Fig.8, Fig.S8, Fig.S11A, Fig.S12
PST25A	29.6 (1.2)	Cationic (-NH2), pHrodo	Fig.4C-F
PST50A	53.8 (2.8)	Cationic (-NH2), pHrodo	Fig.4C-F
PST100A	111.9 (2.1)	Cationic (-NH2), pHrodo	Fig.4C-F
Homemade, see methods	115.6 (34.3)	N/A	Fig.S2

* Nanoplastic particles were purchased from Lab261, Inc.

** S.E.M.s are calculated from at least triplicate independent measures using dynamic light scattering.

***Free dyes were removed from the preparations with desalting columns prior to usage. All concentrated nanoplastics were maintained in 0.1% tween-20 to prevent aggregation of the particles prior to usage in experiments.

Supplementary Methods

Pyrolysis Gas Chromatography and Mass Spectrometry

Frozen frontal cortex brain samples were lyophilized overnight under vacuum. Blood vessels exhibited a leathery texture and were separated easily prior to crushing the remaining tissue into a powder. Approximately 200 µg of the prepared powder for each sample was analyzed through loading the powder to a pyrolysis cup in a first desorption step at 250°C for 5 min to elucidate semi-volatile analytes (e.g., proteins, sugars, lipids), and a pyrolysis step at 600°C to generate pyrolyzates indicative of possible polymer(s) or ions from other heat resistant molecules. All samples were measured in duplicate with similar ion traces obtained. Resulting fragments and rearrangements were analyzed and identified by gas chromatography followed by mass spectrometry, and searched against the NIST 14 Mass Spectral Library and internal reference spectra. Desorb analysis of the samples detected high quantities of cholesterol, and lesser concentrations of other sterols, lipid fragments, and other solvents. A 10 µg polystyrene standard was also analyzed separately to provide signal to noise data for detection limit and rough estimation of polystyrene sensitivities during the pyrolysis step. Typical reproducibility as determined by statistical process control of the measurement system is estimated at about 20% (at 95% confidence level, k~2).

Captions for Auxiliary Supplemental Materials

Auxiliary Raw Data for "Anionic Nanoplastic Contaminants Promote Parkinson's Disease-Associated α-Synuclein Aggregation."
Seventeen tabs, labeled corresponding to the labeled figure panels, provide all raw data used to create the graphs presented in the Figures.
